# Bilateral Morgagni hernia in a Palestinian child with Down syndrome: a rare case report and literature review

**DOI:** 10.1097/MS9.0000000000003961

**Published:** 2025-09-24

**Authors:** Anwar Abu Hetta, Amal M. Shawabka, Bara M. AbuIrayyeh, Bushra Kh. Pujee, Iman M. Alwahsh, Haneen I. Eid, Rahaf W. Thabaineh

**Affiliations:** a Pediatric Assistant Professor- Alia Hospital, Hebron, Palestine; b Faculty of Medicine, Palestine Polytechnic University, Hebron, Palestine

**Keywords:** bilateral diaphragmatic hernia, case report, congenital anomalies, down syndrome, morgagni hernia

## Abstract

**Introduction and Importance:**

Morgagni hernia (MH) is the rarest congenital diaphragmatic hernia, comprising 2–5% of cases, usually right-sided. Bilateral MH is extremely rare, with poorer outcomes, and often involves herniation of abdominal organs into the thoracic cavity. Down syndrome (DS) increases MH risk, but bilateral cases in DS are seldom reported.

**Case Presentation:**

A 14-month-old Palestinian male with karyotype-confirmed DS presented with recurrent respiratory infections. Chest radiography revealed a cystic thoracic mass. Computed tomography confirmed bilateral MH containing the transverse colon.

**Clinical Discussion:**

Bilateral MH in DS is rare and may present with nonspecific respiratory symptoms. Imaging is crucial for diagnosis, and surgical repair prevents serious complications. The patient underwent uneventful open repair, with no recurrence to date.

**Conclusion:**

In DS patients with persistent respiratory distress, bilateral MH should be considered. Early diagnosis and timely surgery can achieve excellent outcomes despite its rarity.

## Introduction

Morgagni hernia (MH) is the most uncommon type of congenital diaphragmatic hernia (CDH), accounting for approximately 2–5% of cases^[1]^. They are frequently found on the right side of the sternum^[2]^. Bilateral defects are extremely rare and can be associated with poorer outcomes, particularly in cases with delayed diagnosis or significant associated anomalies^[3]^. Although severe cases can involve the stomach, liver, and small intestines, MHs allow abdominal contents like the colon and omentum to enter the thoracic cavity^[4]^. There are no specific physical features to support a diagnosis of MH. Patients may present with repeated respiratory distress or inability to thrive in infancy. However, in some cases, MH is identified inadvertently by imaging later in adulthood^[5]^. Congenital cardiac problems, anomalies of the chest wall, and certain genetic syndromes, like Down syndrome (DS), can be linked to certain occurrences of MH. Usually, a transabdominal or transthoracic procedure is used to fix the abnormality^[6]^. Here, we describe a 14-month-old boy, DS, who had persistent respiratory problems. After a suspicious cystic mass was discovered on a chest X-ray, computed tomography (CT) revealed a rare bilateral MH that included the transverse colon. This work has been reported in line with the Surgical Case Report (SCARE) 2025 guidelines^[7]^.

**HIGHLIGHTS**

- Morgagni hernia (MH) is the rarest congenital diaphragmatic hernia – representing only 2–5% of cases, with bilateral MH being exceptionally uncommon and clinically significant.
- Bilateral MH carries high morbidity risks – this rare presentation should be suspected in cases of recurrent chest infections or persistent respiratory distress.
- Down syndrome is a known risk factor for MH – however, bilateral MH in Down syndrome patients remains extraordinarily rare, making this case particularly noteworthy.
- Early surgical intervention is crucial – prompt diagnosis and repair prevent life-threatening complications like bowel obstruction or strangulation.

## Case presentation

We present a case of a 14-month-old Palestinian male infant with DS, diagnosed by karyotyping at birth. He was born preterm at 36 + 4 weeks of gestation, via normal vaginal delivery, with a birth weight of 2960 g.

Shortly after delivery, he developed tachypnea, grunting, and oxygen desaturation. He was subsequently transferred to the Neonatal Intensive Care Unit (NICU) as a case of Transient Tachypnea of the Newborn (TTN). A chest X-ray performed at that time was normal. He spent 13 days in the NICU.

At the age of 8 months, the patient began experiencing recurrent chest infections, which were managed in an outpatient setting with multiple courses of antibiotics.

**Figure 1.**
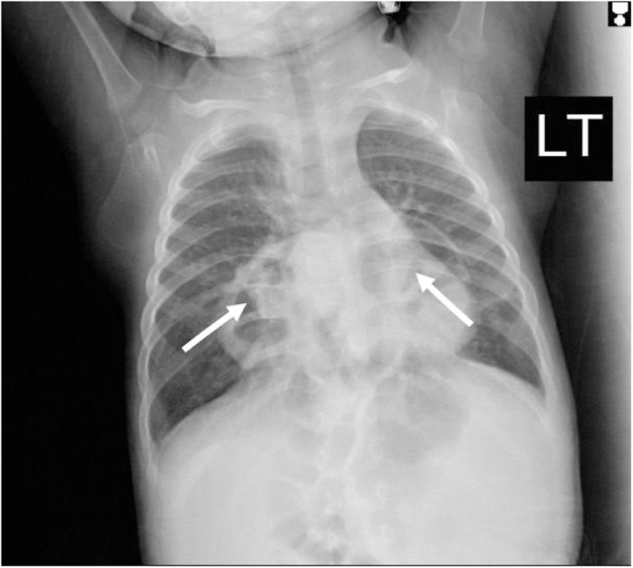
Chest X-ray (AP, supine) showing bilateral, well-defined cystic lesions are seen in the lower thorax (the white arrows), findings are suggestive of bilateral congenital diaphragmatic hernias, likely Morgagni type. No signs of consolidation or effusion.

On April 15, 2024, he was presented to the hospital with nasal congestion, cough, and fever associated with rapid breathing. A chest X-ray was performed and showed a cystic mass (Fig. 1), so he was admitted for further investigation and management.

**Figure 2.**
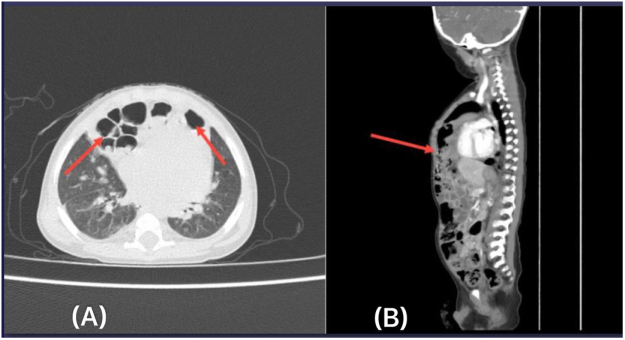
Chest CT scan (A: axial, B: sagittal) showing large bilateral anterior diaphragmatic hernia (with defect about 3 cm), located anterior to the heart, containing segments of the transverse colon, compatible with MH (red arrows), the hernia causing minimal compression on the left ventricle.

On April 15, 2024, a chest CT with contrast was performed to rule out a lung mass (Fig. 2). The imaging revealed right upper and left lower lobe pneumonia, as well as a large anterior bilateral diaphragmatic hernia (with a defect of about 3 cm in sagittal view), located anterior to the heart, containing segments of transverse colon, compatible with MH. A consultation with a pediatric surgeon was subsequently conducted.

Then, the patient was admitted as an elective case for bilateral Morgagni diaphragmatic hernia repair via open technique (laparotomy). The procedure was completed smoothly and without complications. Follow-up after the procedure showed an improvement in the patient’s condition, and there was no evidence of recurrence of hernia up to this date.

## Discussion

Congenital diaphragmatic hernia is a rare embryological defect resulting from incomplete diaphragmatic closure, allowing abdominal viscera to herniate into the thoracic cavity. While the exact pathogenesis remains unclear, approximately 30–50% of cases are associated with chromosomal abnormalities (including trisomies 18, 13, and 21) or other congenital anomalies^[8]^. While different types of CDH occur through different diaphragmatic defects, MH remains the rarest type and occurs in approximately 2–5% of reported cases^[1]^.

A MH arises when the front part of the diaphragm does not form properly during fetal development. This typically happens because of incomplete fusion between the septum transversum and the costal margins. In most cases, it appears on the right side (91%). Hernias on the left are far less common (5%), with only 4% of the cases being bilateral^[9]^. The presence of MHs on both sides, as seen in our patient, represents an unusual and noteworthy finding.

Down syndrome is commonly associated with gastrointestinal anomalies, most notably duodenal atresia (67%), Hirschsprung disease (14%), and tracheoesophageal atresia (10%)^[10]^. Although MH has been reported in this population, bilateral involvement, as seen in our case, is exceptionally rare and has been documented only in a few literatures (Table 1).

**Table 1:** Reported case reports of bilateral MH in Patients with DS

Case report	Age of presentation	Main presentation	Location
Shayba *et al* (2024)^[11]^	1 year and 8 months	Recurrent respiratory infections	Bilateral
Al‑Salem and Khawaher (2002)^[12]^	13 months	Delayed presentation, respiratory distress	Bilateral
Kubiak and Platen (1998)^[13]^	4.5 years	Respiratory difficulties, delayed diagnosis	Bilateral

The clinical manifestations of MH are often variable and subtle, which can delay timely diagnosis. Many patients exhibit nonspecific respiratory symptoms such as chronic cough, wheezing, and recurrent chest infections that can mimic common pediatric conditions like asthma or bronchiolitis^[1]^. In other case scenarios, patients may present with intermittent gastrointestinal symptoms such as abdominal pain, nausea, vomiting, or signs of bowel obstruction, though such presentations are less frequent^[14]^. Importantly, a significant number of MHs are discovered incidentally during imaging performed for other reasons (in up to 50% of cases), especially in asymptomatic individuals or those with vague clinical findings^[1]^. The diagnostic challenge is even greater in syndromic patients, such as those with DS. In patients with DS, recurrent respiratory infections are common and often attributed to underlying hypotonia, immune dysfunction, and structural airway anomalies^[15]^. These baseline features can obscure the recognition of additional pathologies such as MH, leading to delayed imaging and diagnosis. In our case, the nonspecific respiratory symptoms were initially presumed to be part of the patient’s syndromic profile, highlighting the diagnostic challenge and the importance of maintaining a high index of suspicion for anatomical abnormalities when symptoms persist without clear cause.

Imaging plays a central role in confirming the diagnosis of MH. Although chest X-rays can sometimes suggest the presence of bowel loops or air-fluid levels within the thoracic cavity, the findings are often subtle or nonspecific^[16]^. In our patient, definitive diagnosis was done through CT, which provided clear visualization of bilateral anterior diaphragmatic defects along with transverse colon herniation into the chest. CT remains the most reliable imaging modality for identifying the extent and content of the hernia, particularly in unusual or complex presentations where initial investigations may be inconclusive^[5]^.

Surgical repair is the definitive treatment for MH and is generally associated with excellent outcomes. There are different surgical approaches for MH repair, including both open (transabdominal or thoracotomy) and laparoscopic techniques. Multiple studies have demonstrated that laparoscopic repair of MH offers several advantages over the open approach, including shorter operative time, quicker return to feeding, reduced need for postoperative pain management, and shorter hospital stays^[17]^. Importantly, these benefits are achieved without an increase in complications or recurrence rates^[18]^. However, our patient underwent successful surgical correction via an open technique (laparotomy), with marked improvement in respiratory symptoms postoperatively. Early diagnosis and intervention are critical to avoid complications such as bowel obstruction, strangulation, or chronic pulmonary compromise^[1]^.

The limitation of this case report is its nature as a single patient observation, which restricts the generalizability of the findings. Furthermore, we are unable to evaluate late complications or recurrence at this time because long-term postoperative follow-up is still ongoing. Due to the rarity of bilateral MH in patients with DS, there is also limited comparative data in the existing literature to contextualize our findings more broadly.

## Data Availability

The data used to support the findings of this study are included in the article.

## References

[1] Svetanoff WJ, and Rebecca M. Rentea. “Morgagni Hernia.” US National Library of Medicine: StatPearls [Internet] 12 Aug 2024, www.ncbi.nlm.nih.gov/books/NBK557501/. Accessed 24 June 2025.

[2] Leshen M Richardson R. Bilateral morgagni hernia: a unique presentation of a rare pathology. Case Rep Radiol 2016;2016:7505329. 27403367 10.1155/2016/7505329 PMC4923526

[3] Dumpa V, and Chandrasekharan P. Congenital Diaphragmatic Hernia. [Updated 2023 Aug 8]. In: StatPearls, StatPearls Publishing; 2025 Jan. Available from: https://www.ncbi.nlm.nih.gov/books/NBK556076/32310536

[4] Sanford Z Weltz AS Brown J. Morgagni hernia repair: a review. Surg Innov 2018;25:389–99. 29808766 10.1177/1553350618777053

[5] Alrashidi AS Amawi MA Alanazi NO. Morgagni hernia in down syndrome: a case report. Cureus 2023;15:e48019. 38034278 10.7759/cureus.48019 PMC10687593

[6] Daifoladi AA Talemi HG Rezaei MA. Concomitant trans-sternal repair of morgagni hernia and ventricular septal defect in a patient with down syndrome: a case report. Int J Surg Case Rep 2022;92:106911. 35245851 10.1016/j.ijscr.2022.106911 PMC8892097

[7] Kerwan A Al-Jabir A Mathew G. Revised Surgical CAse REport (SCARE) guideline: an update for the age of Artificial Intelligence. Prem J Sci 2025;10:100079.

[8] Chatterjee D Ing RJ Gien J. Update on congenital diaphragmatic hernia. Anesth Analg 2020;131:808–21. 31335403 10.1213/ANE.0000000000004324

[9] Mohamed M Al-Hillan A Shah J. Symptomatic congenital morgagni hernia presenting as a chest pain: a case report. J Med Case Rep 2020;14:13. 31952551 10.1186/s13256-019-2336-9 PMC6969475

[10] Stoll C Dott B Alembik Y. Associated congenital anomalies among cases with down syndrome. Eur J Med Genet 2015;58:674–80. 26578241 10.1016/j.ejmg.2015.11.003

[11] Shayba NA Malkani I Shaikh M. Unusual associations of bilateral morgagni hernia in a child with down’s syndrome: a case report. Inter Surg J 2024;11:1886–89.

[12] Al-Salem AH Khawaher HA. Delayed presentation of bilateral morgagni’s hernia in a child with down’s syndrome. Saudi Med J 2002;23:237–39. 11938406

[13] Kubiak R Platen C Schmid E. Delayed appearance of bilateral morgagni herniae in a child with down’s syndrome. Pediatr Surg Int 1998;13:600–01. 9799386 10.1007/s003830050414

[14] Kumar A Bhandari RS. Morgagni hernia presenting as gastric outlet obstruction in an elderly male. J Surg Case Rep 2016;2016:rjw126. 27432902 10.1093/jscr/rjw126 PMC4948761

[15] Ghezzi M Garancini N De Santis R. Recurrent respiratory infections in children with down syndrome: a review. Children (Basel) 2024;11:246. 38397357 10.3390/children11020246 PMC10888118

[16] Supomo S Darmawan H. A rare adult morgagni hernia mimicking lobar pneumonia. Turk J Surg 2022;38:98–100. 35873743 10.47717/turkjsurg.2022.3978 PMC9278365

[17] Alqahtani A Al-Salem AH. Laparoscopic-assisted versus open repair of morgagni hernia in infants and children. Surg Laparosc Endosc Percutan Tech 2011;21:46–49. 21304389 10.1097/SLE.0b013e318209021f

[18] Young MC Saddoughi SA Aho JM. Comparison of laparoscopic versus open surgical management of morgagni hernia. Ann Thorac Surg 2019;107:257–61. 30296422 10.1016/j.athoracsur.2018.08.021

